# The relationship between childhood adversity and food insecurity: ‘It’s like a bird nesting in your head’

**DOI:** 10.1017/S1368980014003036

**Published:** 2015-01-22

**Authors:** Mariana Chilton, Molly Knowles, Jenny Rabinowich, Kimberly T Arnold

**Affiliations:** 1 School of Public Health, Drexel University, 3600 Market Street, 7th Floor, Philadelphia, PA 19104, USA; 2 Last Mile Health, Tiyatien Health, Zwedru, Grand Gedeh County, Liberia

**Keywords:** Food insecurity, Very low food security, Adverse childhood experiences, Violence

## Abstract

**Objective:**

Adverse childhood experiences, including abuse, neglect and household instability, affect lifelong health and economic potential. The present study investigates how adverse childhood experiences are associated with food insecurity by exploring caregivers’ perceptions of the impact of their childhood adversity on educational attainment, employment and mental health.

**Design:**

Semi-structured audio-recorded in-person interviews that included (i) quantitative measures of maternal and child health, adverse childhood experiences (range: 0–10) and food security using the US Household Food Security Survey Module; and (ii) qualitative audio-recorded investigations of experiences with abuse, neglect, violence and hunger over participants’ lifetimes.

**Setting:**

Households in Philadelphia, PA, USA.

**Subjects:**

Thirty-one mothers of children <4 years old who reported low or very low household food security.

**Results:**

Twenty-one caregivers (68 %) reported four or more adverse childhood experiences, and this severity was significantly associated with reports of very low food security (Fisher’s exact *P*=0·021). Mothers reporting emotional and physical abuse were more likely to report very low food security (Fisher’s exact *P*=0·032). Qualitatively, participants described the impact of childhood adverse experiences with emotional and physical abuse/neglect, and household substance abuse, on their emotional health, school performance and ability to maintain employment. In turn, these experiences negatively affected their ability to protect their children from food insecurity.

**Conclusions:**

The associations between mothers’ adverse experiences in childhood and reports of current household food security should inspire researchers, advocates and policy makers to comprehensively address family hardship through greater attention to the emotional health of caregivers. Programmes meant to address nutritional deprivation and financial hardship should include trauma-informed approaches that integrate behavioural interventions.

Household food insecurity includes two aspects of food security: (i) low food security, which includes multiple indications of food access problems and reduced diet quality; and (ii) very low food security, which indicates that food intake of at least one household member was reduced and eating patterns were disrupted at times during the year because the household lacked money and other resources for food. In 2012, among all US households with children under the age of 6 years, 15868000 individuals (21·5 %), including 8246000 children (22·7 %), lived in households reporting household food insecurity. The number of people living in households with young children under the age of 6 years reporting household very low food security was 4 261 000 (5·8 %). Food security is also calculated at the child level to capture a more severe form of food security in which food hardship is reported to affect children directly. The percentage of individuals in households where there was low food security among children was 11·1 %, representing 4 040 000 children^(^ ^1^ ^,^ ^2^ ^)^.

Regardless of food security status at the child level, food insecurity at the household level has been shown to have major negative consequences for child health and well-being^(^ ^3^ ^)^. Psychosocial stress such as maternal anxiety^(^ ^4^ ^)^, clinical depression^(^ ^5^ ^,^ ^6^ ^)^, social isolation^(^ ^7^ ^)^ and potentially harmful parenting practices^(^ ^8^ ^)^ are also associated with food insecurity among households with children. Additionally, maternal depressive symptoms are strongly associated with food insecurity and with poor child development and behaviour^(^ ^4^ ^,^ ^9^ ^,^ ^10^ ^)^, and depression has been found to mediate the relationship between exposure to domestic violence and food security^(^ ^11^ ^,^ ^12^ ^)^. Overall, stress, anxiety, poor mental health and exposure to violence are strongly associated with food insecurity among adults and children^(^ ^13^ ^–^ ^24^ ^)^.

Most food security studies investigate family characteristics at a singular moment in time; however, some exemplary studies have investigated the causes and characteristics of caregivers’ mental health status retrospectively. For instance, low-income mothers who experienced sexual assault in childhood were over four times more likely to report household-level food insecurity as adults than women who had not been assaulted^(^ ^25^ ^)^. Mothers in persistently food-insecure homes measured over time had significantly higher rates of depression and/or a psychotic spectrum disorder and were more likely to have experienced domestic violence^(^ ^26^ ^)^. Other than these studies mentioned above, lifetime exposures to violence at the household and community level have largely been ignored as potential risk factors associated with household food security. This is despite growing evidence that stress and deprivation during childhood have negative lifetime health and income consequences^(^ ^27^ ^)^. Adverse childhood experiences are associated not only with depression, but also poor school and job performance and drug addiction^(^ ^28^ ^–^ ^31^ ^)^. Additionally, exposure to violence is largely ignored in food security research despite strong evidence that recipients of Temporary Assistance for Needy Families (TANF) and the Supplemental Nutrition Assistance Program (SNAP), programmes meant to support low-income families with cash, work support and nutrition assistance, report high rates of exposure to individual and community violence^(^ ^32^ ^–^ ^34^ ^)^. Conventional efforts to address exposure to violence include venues of health care, education, criminal and juvenile justice, child welfare and supportive housing^(^ ^35^ ^–^ ^41^ ^)^. However, anti-poverty and nutrition assistance programmes are largely excluded from emerging national efforts to create trauma-informed social service systems.

Growing evidence from research in neuroscience indicates that exposure to violence during childhood and adolescence affects financial success in adulthood^(^ ^42^ ^)^. These studies suggest that such exposure may be a factor that distinguishes very-low-food-secure households from low-food-secure households. The relationships between adversity and food security may be explained through mechanisms identified by trauma theory, which has been further substantiated by studies using the Adverse Childhood Experiences (ACE) measure. Adverse childhood experiences such as neglect and abuse, often referred to as ‘toxic stress’^(^ ^43^ ^)^, are associated with major adult diseases such as diabetes, CVD, depression, anxiety and early mortality^(^ ^44^ ^,^ ^45^ ^)^. In addition, recent research identifies how adversity occurring in childhood and adolescence has a decisive impact on behaviours, choices and social relationships that extend into adulthood^(^ ^46^ ^,^ ^47^ ^)^. Experiencing violence, abuse and neglect can result in states of constant heightened aggressive arousal, withdrawing and/or experiencing social isolation, and struggling to keep boundaries associated with normal social and professional behaviour regarding intimacy, safety and security, and job stability^(^ ^35^ ^,^ ^48^ ^,^ ^49^ ^)^. Exposure to adverse experiences in childhood has also been linked to higher rates of worker absenteeism and stress surrounding work and finances in adulthood, indicating strong associations between adverse childhood experiences and financial skills^(^ ^50^ ^)^.

For parents and caregivers of young children experiencing food insecurity, these adult health and behavioural outcomes may hinder their ability to complete their education, find and maintain employment that pays a living wage, and devote the enormous time and energy needed to effectively manage very little income in a way that buffers their children from hunger. The Childhood Stress study utilized quantitative and qualitative methods to identify the pathways through which childhood adversity is associated with household food security among adult caregivers of young children.

## Methods

The Childhood Stress study investigated the circumstances in which caregivers who have experienced severe stress in their early lives come to experience low or very low food security as adults caring for their own children. The Institutional Review Board at Drexel University reviewed and approved the study. Thirty-one participants for the Childhood Stress study were recruited between 2012 and 2014 from families requesting outreach services after participating in the ongoing Children’s HealthWatch–Philadelphia study, a multi-site surveillance study conducted in the Emergency Department of a large Philadelphia children’s hospital. Eligible participants were English- or Spanish-speaking primary caregivers of at least one child under the age of 6 years from households that reported low or very low food security at the household level in response to the US Household Food Security Survey Module (HFSSM). Caregivers were invited to participate in semi-structured interviews for the Childhood Stress study through mailed flyers. Interviews were conducted in participants’ homes, with the exception of seven interviews in which participants requested to meet away from their home environments in another location such as the local library.

The Childhood Stress interview consisted of demographic, quantitative and open-ended questions. The quantitative portion of the interview was carried out first, and included questions on demographics, health status, economic circumstances, public assistance participation, employment characteristics and adverse childhood experiences. The qualitative interview portion that followed was informed by the responses in the quantitative interview, with multiple follow-up questions for clarification and rich description regarding: the quality and characteristics of childhood experiences with hunger, deprivation, abuse and neglect; experiences with education and employment; history of participation in public assistance programmes; and experiences of hunger during childhood, as an adult and among participants’ children.

### Quantitative measures and analysis

The eighteen-item HFSSM was used to measure household and child food security^(^ ^51^ ^)^. In addition to the HFSSM, we included demographic data and federal assistance programme participation status. Caregiver’s report of the child’s overall health status was asked in standard form from the Third National Health and Nutrition Examination Survey and treated as a binary variable (excellent/good *v.* fair/poor)^(^ ^52^ ^)^. To measure caregiver depressive symptoms, we utilized the Kemper three-item screen of maternal depressive symptoms^(^ ^53^ ^)^. The ACE scale is a retrospective ten-item scale that assesses adverse childhood experiences in three domains: (i) physical neglect and abuse; (ii) emotional neglect and abuse; and (iii) household dysfunction, such as having an incarcerated household member, or being a witness to violence against the mother or stepmother (see Table 1). The ACE score (range: 0–10) is based on the cumulative number of affirmative reports of exposure to adverse childhood experiences during childhood^(^ ^44^ ^)^ with good test–retest reliability^(^ ^54^ ^)^.

**Table 1:** Adverse Childhood Experiences survey questions, all answers yes/no

ABUSE
Emotional
(Did a parent or other adult in the household…) Often or very often swear at you, insult you, put you down or humiliate you? OR Act in a way that made you afraid you might be physically hurt?
Physical
(Did a parent or other adult in the household…) Often or very often push, grab, slap or throw something at you? OR Ever hit you so hard you had marks or were injured?
Sexual
(Did an adult or person at least 5 years older than you…) Ever touch or fondle you or have you touch their body in a sexual way? OR Attempt or actually have oral, anal or vaginal intercourse with you?
NEGLECT
Emotional
(Did you often or very often feel that…) No one in your family loved you or thought you were important or special? OR Your family didn’t look out for each other, feel close to each other or support each other?
Physical
(Did you often or very often feel that…) You didn’t have enough to eat, had to wear dirty clothes and had no one to protect you? OR Your parents were too drunk or high to take care of you or take you to the doctor if you needed it?
HOUSEHOLD DYSFUNCTION
Parental separation
Were your parents separated or divorced?
Mother abused
(Was your mother or stepmother…) Often or very often pushed, grabbed, slapped or had something thrown at her? OR Sometimes, often or very often kicked, bitten, hit with a fist or hit with something hard? OR Ever repeatedly hit for at least a few minutes or threatened with a gun or a knife?
Substance use
Did you live with anyone who was a problem drinker or alcoholic or who used street drugs?
Mental illness
Was a household member depressed or mentally ill, or did a household member attempt suicide?
Incarceration
Did a household member go to prison?

Data from the Childhood Stress survey were analysed independently and in comparison with the food security data from the Children’s HealthWatch interview through which participants were recruited. McNemar and Fisher’s exact tests were performed to determine differences in demographic characteristics by household food security. Fisher’s exact test was utilized to ascertain differences in distribution of specific dimensions of the ACE scale according to household and child food security status. We performed Spearman’s *ρ* correlation analyses to examine the association between the cumulative ACE score and the numerical values of household and child food security status for participants in our sample^(^ ^51^ ^)^. While use of quantitative data and statistical analysis in predominantly qualitative studies can offer important complementary information, it can be problematic when it suggests greater generalizability than is warranted by the size and sampling methods^(^ ^55^ ^)^. The quantitative analyses presented herein are not meant to be generalizable, given the small convenience sample, but rather to enhance our internal validity and guide analysis of qualitative results by identifying adverse childhood experiences associated with very low food insecurity.

### Qualitative data collection and analysis

The semi-structured qualitative interview occurred after participants responded to the survey that included the ACE scale and other questions about food security status, public assistance participation and health. Each interview was audio-recorded and lasted between 1·5 and 4·5 h. Qualitative interviews began with developing a timeline of significant life events described by participants. Questions focused on exposure to adverse childhood experiences that were reported earlier in the quantitative portion of the interview, participation in public assistance programmes and food security. Sample questions included: ‘Between the ages of 5 and 10, how do you remember your feelings of safety and security?’; ‘How did your personal experiences with violence [or rape/or abuse, and/or neglect] affect your ability to succeed in school [or continue your education/or your ability to find and keep a good job]?’; and ‘How do these experiences affect the health of your children?’

Interviews were transcribed verbatim and entered into ATLAS.ti, a qualitative software system that assists in management and analysis. System capabilities range from storing and coding narrative qualitative data to organizing transcripts into ‘families’ in order to compare themes across and within groups. Using a grounded theory approach, the authors coded an overlapping set of six interviews to develop a preliminary code set consisting of seventeen major codes and approximately 180 sub-codes (see Table 2). Two authors then coded the remaining transcripts. Themes described here include experiences with neglect, abuse, violence, hunger and household instability, as well as effects of these experiences on educational attainment, employment, and physical and mental health.

**Table 2:** Selected codes from preliminary master code list

Super-code and selected sub-codes (this list does not include all sub-codes)	Number of sub-codes and sub sub-codes
1.Education/school	10
2.Jobs/work/income	9
3.Public assistance	
A. Affects (buffering/more hardship)	12
4.Child welfare authorities	3
5.Hunger/food	10
A. Physical/mental/social sensation	
B. Coping mechanisms	
C. Nutrition	
6.Resilience	1
7.Mental health	12
8.Substance use	16
9.Relationships	16
10.Motherhood, parenting	8
11.Pregnancy	4
12.Intergenerational transmission	12
13.Criminal justice system	5
14.Home, homelessness and housing	11
15.‘The streets’	1
16.Survival	1
17.Violence	51
A. Adverse childhood experiences	
B. Homicide	
C. Suicide	
D. Assault	
E. Captivity	
F. Physical abuse, threats of violence	
G. Community violence	
H. Rape/molestation	
I. Typology (impact; locus; life stage)	

Participants are identified by pseudonym. Some words such as ‘like’ and ‘um’ are omitted for clarity; rephrases and pronoun clarifications that do not change meaning of the quotation are in brackets.

## Quantitative results

Demographic results in Table 3 show that twelve participants reported low household food security and nineteen reported very low household food security. At the child level, sixteen participants reported food security, eleven low child food security and four very low child food security. There were no significant differences among the groups when compared by food security status with the exception of child age (with those reporting very low food security having slightly older children) and race/ethnicity (with more caregivers reporting very low food security identifying as Hispanic or Latina). Twenty-five (81 %) reported that their children were in excellent or good health, while seventeen (55 %) identified their physical health as excellent/good. Twenty-six (86 %) reported maternal depressive symptoms.

**Table 3:** Selected participant characteristics by household food security status; sample of thirty-one mothers of children <4 years old, Philadelphia, PA, USA

	Total (*n* 31)		Low food secure (*n* 12)		Very low food secure (*n* 19)		
	Mean or *n*	sd or %	Mean or *n*	sd or %	Mean or *n*	sd or %	*P* value
Child’s age (months)	27	9	27	9	28	10	0·004**
Caregiver’s age (years)	26·6	15·1	17·6	11·1	32·3	14·7	0·617
Caregiver’s race/ethnicity							
Black/African-American (non-Hispanic)	17	55	10	83	7	37	0·007**
Hispanic/Latina	12	39	1	8	11	58	
Black/African-American and Hispanic	1	3	1	8	0	0	
Biracial	1	3	0	0	1	5	
Employment							
Unemployed	19	61	5	42	14	74	0·130
Employed	12	39	7	58	5	26	
Education							
Some high school	8	26	4	33	4	21	0·806
High school/GED	11	36	4	33	7	37	
Technical school/any college	12	39	4	33	8	42	
Marital status							
Unmarried	29	94	11	92	18	95	1·0
Married	2	7	1	8	1	5	
Mother location of birth							
Born in USA or Puerto Rico	29	94	12	100	17	90	0·510
Foreign born	2	7	0	0	2	11	
Child physical health							
Excellent/good	25	81	12	100	13	68	0·059
Fair/Poor	6	19	0	0	6	32	
Caregiver physical health							
Excellent/good	17	55	8	67	9	47	0·461
Fair/poor	14	45	4	33	10	53	
Caregiver depressive symptoms							
No	5	16	4	33	1	5	0·060
Yes	26	84	8	67	18	95	
Public assistance participation							
TANF	17	55	8	67	9	47	0·461
SNAP	28	90	11	92	17	90	1·0
WIC	25	81	10	83	15	79	1·0
Child food security status at recruitment							
Food secure	16	52	10	83	6	32	0·020*
Low food secure	11	36	2	17	9	47	
Very low food secure	4	13	0	0	4	21	
ACE score							
0–3	10	32	7	58	3	16	0·021*
≥4	21	68	5	42	16	84	

GED, General Educational Development; TANF, Temporary Assistance for Needy Families; SNAP, Supplemental Nutrition Assistance Program; WIC, Special Supplemental Nutrition Program for Women, Infants, and Children; ACE, Adverse Childhood Experiences scale.

Child’s age and caregiver’s age are presented as mean and standard deviation; categorical variables are presented as number and percentage.

* Statistically significant at *P*<0·05.

** Statistically significant at *P*<0·01.

Twenty-one participants (68 %) reported an ACE score of ≥4 and were more likely to report very low food security (*P*=0·021). Severity of household food security was correlated with the number of adverse childhood experiences (Spearman’s *ρ*=0·423, *P*<0·02). The majority of participants, twenty-six (83 %), reported having parents who were separated or divorced, while twelve (39 %) reported physical neglect, the least prevalent of the adverse childhood experiences (Table 4). Reports of physical and emotional abuse were significantly associated with very low food security at the household level and food insecurity at the child level.

**Table 4:** Distribution of types of adverse childhood experiences by household and child food security status; sample of thirty-one mothers of children <4 years old, Philadelphia, PA, USA

			Household food security					Child food security				
	Total (*n* 31)		Low food secure (*n* 12)		Very low food secure (*n* 17)			Food secure (*n* 16)		Food insecure (*n* 15)		
Adverse childhood experience	*n*	%	*n*	%	*n*	%	*P* value	*n*	%	*n*	%	*P* value
Emotional abuse	16	52	3	25	13	68	0·029*	5	31	11	73	0·032*
Physical abuse	16	52	2	17	14	74	0·003**	5	31	11	73	0·032*
Sexual abuse	14	45	4	33	10	53	0·461	6	38	8	53	0·479
Emotional neglect	18	58	4	33	14	74	0·060	7	44	11	73	0·149
Physical neglect	12	39	3	25	9	47	0·274	5	31	7	47	0·473
Parents separated/divorced	26	84	11	92	15	79	0·624	14	88	12	80	0·654
Mother abused	13	42	5	42	8	42	1·0	6	38	7	47	0·722
Household substance abuse	20	65	6	50	14	74	0·255	9	56	11	73	0·458
Household mental illness	19	61	5	42	14	74	0·130	9	56	10	67	0·716
Household incarceration	15	48	6	50	9	47	1·0	10	63	5	33	0·156

* Statistically significant at *P*<0·05.

** Statistically significant at *P*<0·01.

## Qualitative results

The relationship between adversity and food insecurity is described in two ways: (i) through a case example to demonstrate the cumulative effects of adversity; and (ii) by focusing on participants’ descriptions of emotional and physical abuse and neglect, which were shown to be significantly associated with very low food security.

### Multiple adverse childhood experiences in relation to food security: a case example

**Fig. 1:**
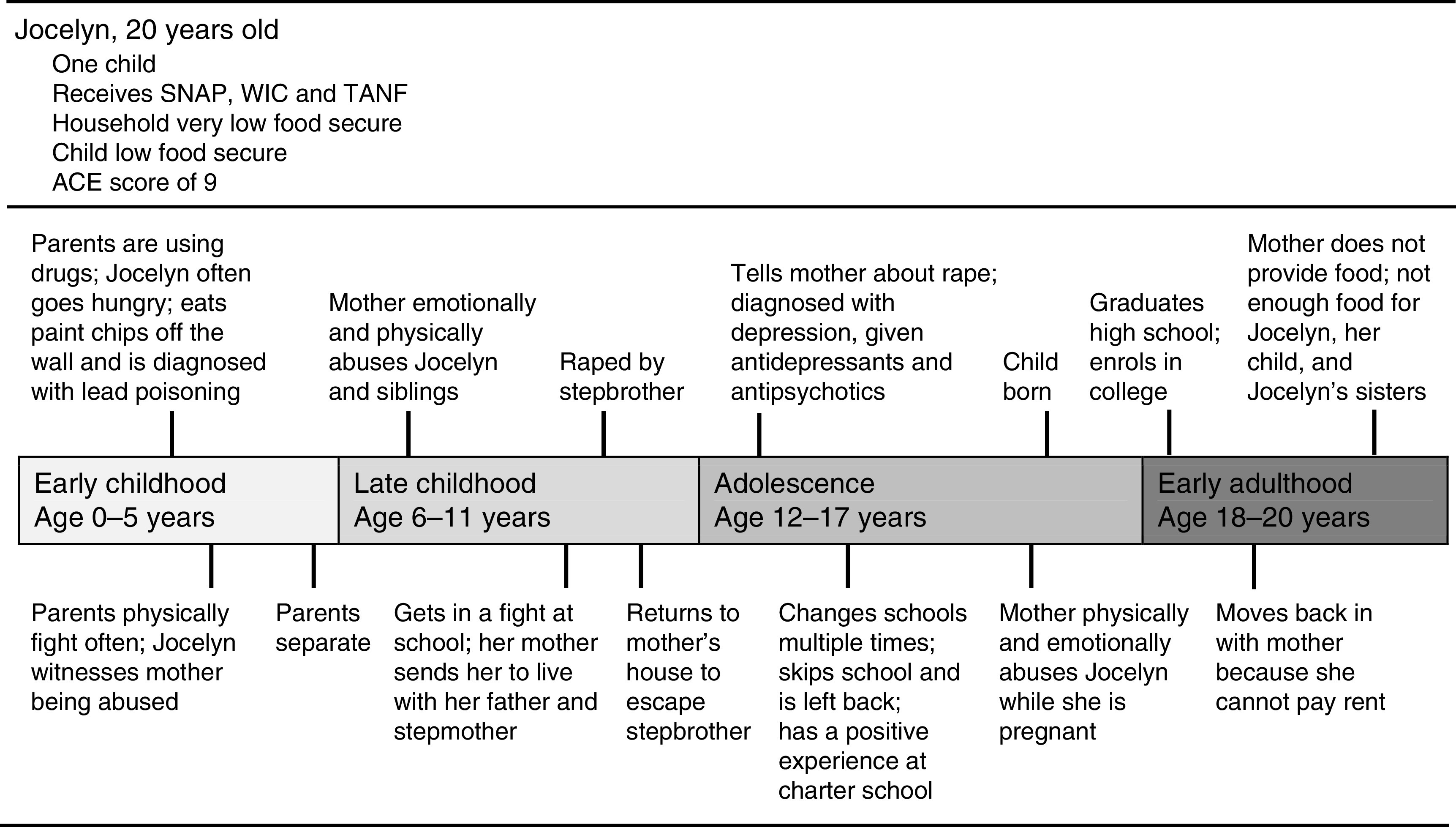
Sample timeline (ACE, Adverse Childhood Experiences scale; SNAP, Supplemental Nutrition Assistance Program; WIC, Special Supplemental Nutrition Program for Women, Infants, and Children; TANF, Temporary Assistance for Needy Families)

Adverse experiences described by the participants, reflecting the interrelatedness of these experiences and a strong relationship between the number of adverse childhood experiences and health and behavioural outcomes as described in the literature^(^ ^44^ ^,^ ^56^ ^–^ ^58^ ^)^, were most devastating when clustered together. As an example, we highlight pivotal experiences described by Jocelyn, a 20-year-old mother of one reporting household very low food security/child low food security, with an ACE score of 9 (see Fig. 1). She described experiences of hunger in her early childhood growing up with parents addicted to drugs:

‘We barely had food. I don’t even know if food stamps existed. They probably did, but my mom worked at [a fast-food restaurant] – not to mention the fact that she was still getting high […] We was always hungry. The only time I’ve learned really eating is when my dad used to drop us off at this lady [name omitted] down the street. She used to babysit us. That’s the only time we really ate. […] In the morning we had to have oatmeal. Before she sent us back with our dad, we used to have cut up hot dogs and baked beans. So that’s the only time we would eat. There was no going to the store. I didn’t know going to the store until I was, like, 8.’

Jocelyn described herself as hungry constantly in the early years of her life. She described being so hungry that she ate paint chips off the wall only later to be diagnosed with lead poisoning. She described getting into fights at school, culminating in an incident that involved the police. As a result, her mother sent her to live with her father and stepmother:

‘Then [when I was] around 10 she got tired of me. She kept saying I was, like, bad; something was wrong with me. So she just finally sent me to my dad and while I was there I got a stepbrother. And, basically, it started with him touching me and then he had sex with me. I was still 10 but it was like two months into me being there. A month later I just, like, said that I didn’t want to […] do nothing. I just started acting out. But like besides [my stepbrother], that was like the best part of my life, like besides everything else […] I got taken care of. I always had something to eat.’

Afraid to tell her father and stepmother about being raped by her stepbrother, Jocelyn chose to go back to her mother’s house, where she continued to be abused and neglected. She eventually told her mother and a counsellor that she had been raped, and was hospitalized for depression at the age of 13. Her father never acknowledged or apologized for the abuse she suffered under his care. She continued to struggle in school, finding herself in many fights because of difficulty managing anger. She described further incidents where her mother physically abused her during her pregnancy with her son, who was born when Jocelyn was 17. Jocelyn was fired from the only job she ever held, as a cashier, when she tried to rearrange her hours to attend college. Unable to pay rent without a job, Jocelyn was again living with her mother at the time of the study interview. She described continuous emotional abuse from her mother and the added responsibility to feed her younger siblings, who were often left in her care without food because of her mother’s drug use. These circumstances left her skipping meals and struggling to stretch her own food stamps to ensure that her younger siblings ate.

This accumulation of adverse experiences in Jocelyn’s life, including household drug abuse, physical and emotional neglect, as well as sexual, physical and emotional abuse, was common to a majority of participants reporting very low food security.

### Emotional abuse and neglect

On the ACE survey, emotional abuse is documented if respondents affirm that an adult in the household ‘often or very often’ swore at them, insulted them, humiliated them or acted in a way that made them fear for their physical safety. Strongly related is emotional neglect, where the respondent feels that no one in her family loved her or made her feel important, or that her family was not supportive of each other. Participants described emotional abuse in connection with poor performance in school and poor mental health. Naitana (household very low food security/child low food security; ACE score of 9), a mother of two, immigrated with her mother from Haiti at age 4 and, after being held at Guantanamo Bay with other refugees, was placed into foster care at age 12 when her mother died from AIDS. After experiencing racism and discrimination when placed with an Amish foster family, she cycled through three other families before running away from home to stay with friends after being raped by her foster mother’s husband at age 14. She described trouble focusing in school, saying that deep depression and suicidal thoughts kept her from wanting to learn. She explains:

‘I could not focus on nothing. And I feel like, okay, why should I learn this and like, why? Why? I’m not going to live for long. I’m going to kill myself, because I just don’t want to live, because what’s the point? Ain’t nobody love me, ain’t nobody going to ever love me the way my mother did. So I don’t want to learn, I don’t want to do this.’

Naitana’s struggles in school were compounded by a learning disability that went undetected as she passed through multiple schools moving between foster homes. Friends and teachers helped her to graduate from high school, but she felt unprepared for the workplace. Currently, she has difficulty finding a job that pays enough to support her family.

Participants also described low self-esteem and a foreshortened sense of possibility as a consequence of emotional abuse and neglect. Tamira (household very low food security/child low food security; ACE score of 9), a 22-year-old mother of one, was abandoned by her mother at the age of 5, abused and raped at the age of 6 by the members of the family with whom she was left, and was emotionally and physically abused by her grandmother who took her in at the age of 7. Tamira described depression, difficulty managing anger and outbursts, and a suicide attempt in her childhood. Her grandmother kicked her out of the house at age 15 and she describes being effectively homeless, living ‘house to house’ ever since. Despite these experiences, she was able to graduate from high school at age 21, but struggles to find work. She described the effects of childhood experiences on her self-esteem and her job prospects, and therefore on her ability to provide for her daughter:

‘If a person always says you’re nothing; you’re nothing. Then for a while I used to think I’m not anything. So maybe that’s how I don’t have a job, because I’m thinking I’m nothing. I’m not ever going to have a job. I’m not going to be shit, like my grandma said. So it’s like maybe that’s a part of how I don’t have a job or I couldn’t finish school […] Because I can’t find a job I cannot feed my daughter. How am I supposed to? I cannot buy her what she needs.’

The emotional adversity experienced in childhood carried forward into adulthood, since participants who described feeling unloved and unsupported by family in childhood were often unable to rely on family for social support as adults. This means that common coping mechanisms described here and in other research, such as borrowing food or money from family or sending children to family’s houses to eat^(^ ^59^ ^)^, were unavailable or came with a toll on safety and mental health.

### Physical abuse and neglect

The ACE survey characterizes physical abuse as often or very often being pushed, grabbed, slapped or having something thrown at you, or being hit hard enough to have marks or be injured. Physical neglect is described as not having enough to eat, having to wear dirty clothes and having no one to protect you, and/or inadequate care related to substance abuse of a parent or caregiver. Emilia (household very low food security/child low food security; ACE score of 6), a 35-year-old mother of four, described being physically abused by her mother:

‘My mom broke my teeth when I was little. She hit me with a curling iron and she chopped my teeth off of my face […] She was mad, because she had came home when I was cleaning… I had ran away, so I lost my room ’cause I ran away. I come home and I was cleaning my sister’s room and it was late.’

Emilia dropped out of school in ninth grade after missing days to care for younger siblings while her mother worked, and ran away from home for good at the age of 17. Escaping homelessness, she moved in with a boyfriend, became pregnant and still struggled to have enough money for food. During her second pregnancy, she turned to her estranged father for money for food. This exposed her to more neglect and emotional abuse, exacerbating her feeling of social isolation:

‘If you asked [my father] for five dollars, he would run from you […] I was pregnant, I was hungry, I was working, I had no money. I had spent all of my money to get catch some buses back and forth all the time. I went over there to ask him for five dollars for food. He just goes berserk and I’m over here like, “Oh”, in tears, “I’m really hungry.” This guy doesn’t even care.’

In many of the interviews experiences of physical neglect and substance abuse were often captured together. Taleya (household low food security/child food security; ACE score of 9), a 39-year-old mother of two, described the effects of her own mother’s drug use on her ability to care for Taleya and her siblings when they were young:

‘That’s when crack started. You know, freebasing or whatever they wanted to call it back then […] She would go to work all the time and she’d just leave the food stamps, because all of the responsibility was on me. I was about maybe 11. You know, and I had my two younger brothers, so we pretty much took care of ourselves. […] We always had the food stamps, until, I guess, her addiction got worse, and then it became… there was nothing in refrigerator, nothing in the cabinets.’

Taleya’s experience is echoed by several participants, who described the added responsibility for feeding themselves as the result of household drug abuse. Karina (household very low food security/child food security; ACE score of 7), a 35-year-old mother of three, described the effects of her stepfather’s drug problem on her mother’s ability to provide for her:

‘Hunger. It was always a hunger issue in the household because of the fact that my mom would get paid and he would take her money. So it was always me going over to my auntie’s house to eat. So it’s like it was always a hunger issue because my stepfather and his ways […] He was a drug addict. He sold drugs. He stole from mother. He was a real violent man, a real violent person.’

Experiences with physical adversity, even once resolved, have lifelong impacts. Claudia, a 22-year-old mother of one (household very low food security/child low food security; ACE score of 9), described how childhood experiences with physical neglect and hunger affect how she cares for her son. After her parents divorced and her mother abandoned the family, her father began drinking heavily, neglecting Claudia and her sisters. When she was 15, Claudia’s father got into an altercation with Claudia’s sister that culminated with him punching Claudia in the face. Her mother, who suffered from severe depression, was nowhere to be found. Consequently, Claudia ran away from home. She lived with a group of neighbourhood teens in an abandoned house and then with an older sister who struggled with mental illness, during which time she experienced chronic hunger. Now, as a mother of a young child, she described how she continued to skip her own meals and live on a very low budget to ensure that her son does not experience the hunger she felt as a teenager. She recently lost her job after taking off multiple days to care for her son, who is asthmatic. Her drive to protect her son from the type of neglect she experienced prevents her from being able to care for herself, which places her in a precarious situation:

‘No matter how good I am, I have to still fight in order to have more. Because it all falls on [my son], because I don’t want him to live through it. I know how hard it was on me. I know how much my stomach hurt from the hunger, how much my body ached, having pains and not having the medication for it, you know? […] The hunger, the pain, the depression – it always comes back. It’s like a bird nesting in your head.’

Even if she were not struggling financially as she is now, Claudia explained, she still feels very troubled by her experiences with hunger. As she explained, ‘it affects the ability to be really happy. Because put it like this: it haunts me.’

## Discussion

Analysis of both qualitative and quantitative results suggests a strong relationship between exposure to adverse childhood experiences and household food insecurity. While the quantitative results should be considered with limited confidence because of small sample size, the distribution of adverse childhood experiences among caregivers reporting very low food security demands considerable pause. Prevalence of emotional and physical abuse (52 %) in this sample is strikingly higher than what is reported for the general population of Philadelphia^(^ ^60^ ^)^, where prevalence rates of emotional and physical abuse are lower (33·2 % and 35 %, respectively). Rates of adverse childhood experiences in our sample are also considerably higher than rates reported in the original Kaiser study with over 17 000 patients in San Diego^(^ ^44^ ^,^ ^60^ ^)^, where 6·2 % reported ACE score of ≥4 compared with nearly two-thirds in our sample.

This sample of food-insecure families has high prevalence of exposure to adversity during childhood, and qualitative descriptions of the relationship between food insecurity and childhood adversity are explicit. The level and breadth of vulnerability stretches beyond economic deprivation to include depression and emotional problems that directly affect education and employment. Among the mothers reporting very low household food security, there were significant experiences that reflected depression and social isolation of the caregiver, both in the past and currently, corroborating previous studies indicating that these are primary factors associated with food insecurity^(^ ^4^ ^,^ ^7^ ^,^ ^12^ ^,^ ^61^ ^–^ ^63^ ^)^. The breadth of experiences described by participants also corroborates recent studies that have recommended expansion of the ten-item ACE questionnaire to include experiences affecting low-income urban communities that document exposure to community violence and participation in foster care^(^ ^49^ ^,^ ^60^ ^)^.

Additionally, the qualitative reports of exposure to violence, trauma and neglect reflect how such experiences continue to affect people into adulthood and as they attempt to care for their own children. These experiences are reflected in their reports of getting into excessive fights in school, or quitting jobs over seemingly small infractions. Both have an impact on financial stability. While public assistance programmes help mitigate the effects of economic deprivation, they must also integrate services that address trauma-related challenges affecting school and job performance and emotional well-being. Studies indicate that public assistance participants, particularly those exposed to abuse and neglect, experience programmes as punitive and disempowering^(^ ^64^ ^,^ ^65^ ^)^. The US Substance Abuse and Mental Health Services Administration has identified best practices for trauma-informed systems to recognize and address widespread exposure to trauma and violence among those seeking support from social service agencies^(^ ^41^ ^)^. Promising interventions from sectors leading this effort, including health care and education, should be adapted for public assistance systems to recognize this exposure and adopt policies that avoid re-traumatization and promote participant safety and agency. Additionally, screening participants for past and current exposure to violence and abuse and appropriate referral to mental health and/or intimate partner violence services is essential.

The analyses conducted in the present study are limited in their generalizability due to the small convenience sample. Recruitment of participants through a hospital emergency department may introduce a selection bias toward participants with higher exposure to violence. The recruitment flyer described that participants can share experiences with ‘stress in childhood’, which may have biased participation toward those interested connecting their childhood experiences with their current circumstances.

Additional research on the relationship between adverse childhood experiences and food insecurity with larger and more representative samples is necessary. Further research into the long-term cognitive, behavioural and social impacts of trauma is also important to more fully explain the causal pathways that connect childhood exposure to adversity with adult economic and food insecurity.

## Conclusion

The deprivation among households with young children reporting primarily very low household food security is related to reports of exposure to adverse childhood experiences of the mother. These results prompt consideration of how nutrition assistance programmes are integrated with other assistance programmes, such as nurturing behavioural health programmes and stabilizing programmes such as housing and child-care subsidies. For families with young children reporting very low food security, public assistance programmes must ensure families are able to meet basic needs beyond cash and food allotments. Families must also have safe places to live and to care for their children, behavioural health support, and opportunities to develop positive social relationships to remedy social isolation and the emotional distress that ‘nests in the head’. Overall, families reporting very low food security need far more support than they currently receive, and this support must take into account their exposure to trauma and violence so that caregivers can take advantage of opportunities to advance their educations, find and maintain employment, and attain economic security.
